# LoRDEC: accurate and efficient long read error correction

**DOI:** 10.1093/bioinformatics/btu538

**Published:** 2014-08-26

**Authors:** Leena Salmela, Eric Rivals

**Affiliations:** ^1^Department of Computer Science and Helsinki Institute for Information Technology HIIT, FI-00014 University of Helsinki, Finland and ^2^LIRMM and Institut de Biologie Computationelle, CNRS and Université Montpellier, 34095 Montpellier Cedex 5, France

## Abstract

**Motivation:** PacBio single molecule real-time sequencing is a third-generation sequencing technique producing long reads, with comparatively lower throughput and higher error rate. Errors include numerous indels and complicate downstream analysis like mapping or *de novo* assembly. A hybrid strategy that takes advantage of the high accuracy of second-generation short reads has been proposed for correcting long reads. Mapping of short reads on long reads provides sufficient coverage to eliminate up to 99% of errors, however, at the expense of prohibitive running times and considerable amounts of disk and memory space.

**Results**: We present LoRDEC, a hybrid error correction method that builds a succinct de Bruijn graph representing the short reads, and seeks a corrective sequence for each erroneous region in the long reads by traversing chosen paths in the graph. In comparison, LoRDEC is at least six times faster and requires at least 93% less memory or disk space than available tools, while achieving comparable accuracy.

**Availability and implementaion**: LoRDEC is written in C++, tested on Linux platforms and freely available at http://atgc.lirmm.fr/lordec.

**Contact:** lordec@lirmm.fr.

**Supplementary information:** Supplementary data are available at *Bioinformatics* online.

## 1 INTRODUCTION

Sequencing, the determination of DNA or RNA sequences, now belongs to the basic experiments in life sciences. Compared with the Sanger method, the so-called next-generation sequencing technologies (of the second, third or even fourth generations) have drastically lowered its cost and increased its efficiency, making genome-wide and transcriptome-wide sequencing feasible. Numerous types of ‘omics’ experiments, beyond *de novo* genome sequencing and assembly, have been invented and rely on high-throughput sequencing.

All currently available technologies produce reads that represent only a piece of the target molecule sequence. Processing these reads requires aligning them against other sequences: for instance, while mapping them against a reference genome, or when computing overlaps among reads during assembly. Optimal, and sometimes suboptimal, alignments are retained for further analysis. The strength of an alignment (and hence its usefulness) is mostly controlled by two factors: its percentage of identity and its length. Clearly, errors introduced during the sequencing process, sequencing errors, blur the signal in an alignment by introducing mismatches or by breaking it into shorter ones. Weaker alignments may not pass subsequent filters and are lost for downward analyses. The finer the analysis, the higher the necessity to capture the information available in all alignments: for instance, when trying to bridge a gap in a less covered region of genome during assembly, or to reconstruct the sequence of a less expressed RNA. To counteract sequencing errors, error correction algorithms have been found effective for *de novo* assembly (Salzberg *et al.*, 2012), and so they are often incorporated in assembly pipelines [see e.g. Euler SR (Chaisson and Pevzner, 2008), ALLPATHS-LG (Gnerre *et al.*, 2011) and SOAPdenovo2 (Luo *et al.*, 2012)].

### 1.1 Related works for second-generation sequencing

In the case of long sequences (Sanger or PacBio reads), algorithms compute multiple alignments of the reads and call a consensus sequence to correct erroneous regions. Alignment computation has the inconvenience of long running time and parameter dependency (Salmela and Schröder, 2011). In the case of second-generation reads, meaning larger input size and modest error rates, the key idea is to exploit the coverage of sequencing. One distinguishes erroneous from error-free substrings by counting their number of occurrences in the read set. With a sufficient coverage, it is possible to compute a minimal threshold such that, with high probability, each error-free *k*-mer appears at least that number of times in the read set. A *k*-mer above/below the threshold is qualified as solid or weak, respectively. This idea is exploited in second-generation assembly programs based on De Bruijn Graphs (DBG), where only solid *k*-mers form the nodes of the DBG (e.g. Zerbino and Birney, 2008), or during mapping against a reference to distinguish erroneous positions from biological mutations (Philippe *et al.*, 2013). Many current error correction algorithms for second-generation sequencing (Illumina, Roche, or Solid) adopt this counting strategy, also called spectral alignment (Chaisson *et al.*, 2004; Pevzner *et al.*, 2001): one computes the spectrum of solid *k*-mers and corrects each read by updating each weak *k*-mer with its closest solid *k*-mer. Implementation relying on hash tables is well adapted to *k*-mers (i.e. to substrings of fixed length), while approaches based on more flexible indexes of the reads (e.g. suffix trees or suffix arrays) can correct substrings of different lengths (Salmela, 2010; Schröder *et al.*, 2009). Spectral alignment-based approaches are more efficient and scalable than alignment-based ones, and adapted to low error rates. Most recent work on error correction has concentrated on correcting Illumina reads where substitutions is the dominant error type, and so the more challenging problem of correcting insertions and deletions is addressed only by a few works (Salmela, 2010; Salmela and Schröder, 2011). For a survey on error correction methods for second-generation sequencing, see Yang *et al.* (2013).

### 1.2 Related works for PacBio reads

PacBio SMRT sequencing is characterized by much longer reads (up to 20 Kb) and much higher error rates (>15% Koren *et al.*, 2012), and poses a much harder challenge for error correction. However, sequencing errors seem to be uniformly distributed, independent of the sequence context and skewed toward insertions, to a lesser extent deletions. For simplicity, we call PacBio reads, long reads (LR), and other second generation reads, short reads (SR). To address this challenge, two approaches have been proposed: **self correction** using only LR, or **hybrid correction** of LR using libraries of SR. Self correction, alike Sanger correction tools, computes local alignments between LR [with BLASR (Chaisson and Tesler, 2012)] for building multiple alignments and then calls a consensus. It has been implemented in a non-hybrid assembler, HGAP, and experimented on bacterial genomes (Chin *et al.*, 2013). Hybrid correction exploits the higher quality and coverage of SR libraries, which give rise to stronger alignments, to align these on LR and correct the latter by calling consensus sequence from a multiple alignment. This strategy, found in assembler AHA (Bashir *et al.*, 2012), and in correction programs LSC (Au *et al.*, 2012) and PacBioToCA (Koren *et al.*, 2012; which has been incorporated in the Celera assembler), achieves similar accuracy on bacterial genomes than a non-hybrid method, but has also proved able to operate on eukaryotic genomes and transcriptomes (Au *et al.*, 2012; Koren *et al.*, 2012).

### 1.3 Genome finishing, scaffolding and limitations of long read correction methods

Recently, two proposals [PBJelly (English *et al.*, 2012) and Cerulean (Deshpande *et al.*, 2013)] have adopted an intermediate strategy for genome scaffolding or finishing: in addition to LR, they take as input either a partially assembled genome or an assembly graph generated with SR data. Contigs are mapped to LR, which serve as the basis to complete/fill the assembly gaps or order the contigs into a scaffold. Deshpande *et al.* (2013) justify their strategy by the time, memory and disk requirements of current LR correction programs, ‘which requires high computational resources and long running time on a supercomputer even for bacterial genome datasets’. Current correction programs seem not to take full advantage of sequence indexing data structures to speed up the correction (Navarro and Mäkinen, 2007).

### 1.4 Contribution

**Fig. 1.**
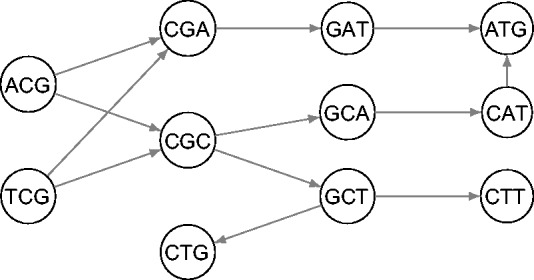
An example of short read DBG of order *k* = 3. For simplicity reverse complement *k*-mers are ignored

Considering the limitations of LR correction programs and the high error rates in PacBio reads, we propose here a new hybrid correction algorithm aiming at more efficiency. It first builds a DBG of the SR data, and then corrects an erroneous region within an LR by searching for an optimal path within the DBG. The sequence of the overlapping *k*-mers along the path provides a corrected sequence for that region. Taking advantage of recent developments in compact representation of DBG [Fig. 1, Chikhi and Rizk (2012); Salikhov *et al.* (2013)], we develop a program, called LoRDEC (Long Read DBG Error Correction), that allows correcting a dataset of typical size on common computing hardware. We compare our program with state-of-the-art methods and find that it provides an equal accuracy with low memory usage and reasonable running times.

## 2 METHODS

### 2.1 Overview

The rationale behind a hybrid correction algorithm is to use a set of high-quality reads to correct a second set of reads suffering from a higher error rate. Typically, a reference set of Illumina, 454, or PacBio CCS SR with low error rate will help correcting long PacBio RS reads. As both sets are assumed to come from the same library, the goal is to convert the sequence of an erroneous region in a long read into the sequence that could be assembled from the SR in that region of the molecule, while keeping the length of LR. Our program, called LoRDEC, takes as input the SR, the LR and an odd integer *k*. Now, our approach is to find, for each erroneous region of an LR, an alternative sequence by traversing appropriate paths in the DBG of the SR. However, SR also contains errors. To avoid introducing erroneous bases during the correction, we filter out any *k*-long substring, termed a *k*-mer, that occurs less than *s* times within the SR [as done in second-generation assemblers (Chikhi and Rizk, 2012; Salikhov *et al.*, 2013; Zerbino and Birney, 2008)], where *s* is set by the user. With our terminology, we keep only solid *k*-mers.

LoRDEC first reads the SR, builds their DBG of order *k* and then corrects each long read, independently, one after the other. The DBG is the graph underlying most second-generation assemblers (e.g. Velvet, Minia). Each solid *k*-mer found in the SR makes a node in the DBG, and a directed arc links a node *f* to a node *g* if the *k*-mer of node *f* overlaps that of *g* by *k* − 1 positions. Figure 1 shows an example of a DBG. As usual in the DBG used for assembly, because the strand of reads are unknown, a node represents a *k*-mer and its reverse complement *k*-mer, and the notion of arc is extended to ensure that two nodes/*k*-mers can overlap each other on the same strand. For instance, a *k*-mer *acgta* would be linked to *k*-mer *cgtat* by an arc. Clearly, a path, i.e. a series of arcs, from one node to another represents a nucleotidic sequence, and between two nodes, say *f* and *g*, there may be none, one or several paths. Typically, assembly programs output the sequence along non-branching paths as contigs. For storing the DBG, we use the memory-efficient GATB library (http://gatb-core.gforge.inria.fr), which allows to traverse any path in the graph and to get the sequence of any node. GATB uses Bloom filters to store the DBG and additionally records those false-positive *k*-mers that are adjacent to *k*-mers in the DBG, which allows traversing only solid *k*-mers if the traversal starts at a solid *k*-mer. However, we use the DBG to determine whether a *k*-mer in an LR is solid and, therefore, GATB can report false positives. We found that if we additionally require that for a *k*-mer to be considered solid, it must also have at least one incoming and at least one outgoing arc, only a small fraction (e.g. 0.03% in the *Escherichia coli* dataset) of the reported solid *k*-mers are false positives.

**Fig. 2.**
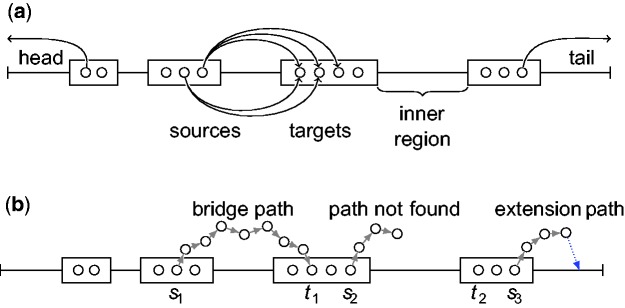
Long read correction method. (**a**) A long read is partitioned into weak and solid regions (respectively, lines and rectangles) according to the short read DBG. Weak regions starting or ending the long read are called the *head* or the *tail*, respectively, while other weak regions are *inner regions*. Circles in solid regions represent *k*-mers of the DBG. *k*-mers around a weak region serve as source and target nodes to search paths in the DBG. Several source/target pairs are used for each weak inner region. (b) On the second inner region, a *bridging path* between nodes *s*_1_ and *t*_1_ is found in the DBG to correct this region. On the third region, the path search fails to find a path between nodes *s*_2_ and *t*_2_. For the tail, an *extension path* is sought and found from node *s*_3_ toward the end. Once found, the corrective sequence of the path is aligned to the tail to determine the optimal substring (thick dotted arrow)

Consider the *k*-mers of a long read starting at position 1,2,3, … : some *k*-mers belong to the graph and are solid, while others do not and are weak. Basically, solid *k*-mers are expected to be correct, while weak ones suspectedly include sequencing errors and require a correction. Solid *k*-mers are entry points in the DBG, and LoRDEC corrects a region made of weak *k*-mers by finding the best path in the DBG between the solid *k*-mers bordering this region. Sometimes, an LR has no solid *k*-mer, in which case, LoRDEC marks it as such in the output and skips it. Our results show that only short erroneous reads (<1500 nucleotides) completely lack solid *k*-mers (data not shown). As the goal of PacBio sequencing is to get long reads and thus our goal is to yield long and correct sequences, we decided to filter those reads in the present version of LoRDEC. In the remaining LR, at least one *k*-mer is solid: consequently, two alternative situations occur for a weak region: either it is located at one end of the LR and only one solid *k*-mer is bordering it, we call these a head or a tail region, or it is an inner region surrounded by a run of solid *k*-mers on each side. Weak regions are shown in Figure 2a. Our algorithm uses two distinct procedures to correct a head/tail or an inner region (see below).

The algorithm for correcting one long read is illustrated in Figure 2 and summarized as follows. For each pass over a long read, we apply the head/tail correction procedure to the left-most (head) and right-most (tail) weak regions, then we loop over the sequence, select pairs of solid *k*-mers and, for each, launch the correction procedure for the weak region between them. Each call for a correction procedure modifies the sequence on-the-fly, and thus turns weak into solid *k*-mers. LoRDEC performs two passes over the read, one in each direction. First, on-the-fly correction generates new solid *k*-mers, which serve as starting nodes in the next pass; second, because of repeats in the sequence, the search for a path can proceed to different parts of the graph depending on which end of the region it is started from. Thus, it is worth trying two passes.

### 2.2 Correction of inner weak regions

An inner region is bordered by a run of solid *k*-mers on each side. The procedure takes as input the source and target solid *k*-mers, the region sequence and a maximal branching limit. Solid *k*-mers serve as source and target nodes in the DBG, and any path between these nodes encodes a sequence that first, can be assembled from the SR, and second, it starts and ends with the appropriate solid *k*-mers. Thus, the region sequence could be corrected with the sequence of any such path. Our criterion to choose among several such paths is to minimize the edit distance between the path and the region sequence. Now, several solid *k*-mers can serve as source and target for the search. The criterion of solidity with which we filter erroneous *k*-mer is not perfect: some solid *k*-mer may still be erroneous. With such *k*-mers the path search may fail or result in a path sequence that is far from optimal. To avoid being trapped in such local minima, we consider not only one but several pairs of (source, target) *k*-mers around each weak region.

For this, we loop over the inner solid *k*-mers of the read and consider each as a source. For each possible source, we consider *t* downward solid *k*-mers as targets (by default *t* is set to 5), and filter out some exclusive cases, depending on whether the source and target *k*-mers

1. belong to the same run of solid *k*-mers: the region is assumed to be correct, and no path is searched for;
2. overlap: a tandem repeat likely creates this overlap region or a *k*-mer is falsely solid, we skip this case;
3. are too distant from each other in the read: the dynamic programming (DP) matrix to compute the minimal edit distance would require too much memory, and the likelihood to find a path would be low: we necessarily skip this case.

In all other cases, we search for an optimal path between a source and target solid *k*-mers. With this manner of selecting source/target pairs, we ensure that several pairs bordering a weak region will be considered. If a path between a source and a target is seen as a bridge over a weak region, then alternative source/target pairs may form distinct bridges that cross over that region of the read. All bridges found along the read form a directed graph: the **path graph**. The solid *k*-mers build its nodes, and each path found is an arc between the source and target *k*-mers. The arcs are weighted by the edit distance between the region sequence and the found path. The path graph construction is thus intermingled with the inner region correction.

To seek an optimal path in the DBG for a selected source/target pair, we perform a depth-first search traversal of possible paths between the source and target, and compute at each step (node wise) its minimal edit distance with the region sequence in a DP matrix. The exploration of a path stops when reaching a dead end in the graph, the target *k*-mer or whenever the minimal edit distance of any extension of the path would exceed maximum allowed error rate. The overall search is aborted whenever the number of paths encountered exceeds the branching limit. In the end, if at least one path was found, we record the path and its edit distance as an arc between the two *k*-mers in the path graph, which we defined above. Otherwise, if the search has failed at all trials with the current source *k*-mer, we add a dummy arc to the path graph: an arc between the source and the next solid *k*-mer weighted by an edit distance equal to the region length. This ensures that a path from the first to the last solid *k*-mer always exists.

### 2.3 Head or tail region correction: searching for a best extension

Correcting the head or the tail of a read is a symmetrical procedure, so we describe it for the tail. A tail is a nucleotidic region made of weak *k*-mers, preceded by at least one solid *k*-mer. The procedure takes as input the solid *k*-mer node as a source node in the DBG, the tail sequence and a branching limit. Unlike for an inner region, we lack a target *k*-mer, and thus need another criterion to stop visiting a path. The procedure seeks for any path that allows correcting a prefix of the tail, and optimizes node-wise the prefix length and the edit distance between the current path and the current prefix of the tail. It uses a depth-first search and explores paths until their edit distance becomes too large, or until reaching either a dead end in the DBG or the end of the tail.

Finally, because the procedure optimizes the prefix length, it tends to extend the search beyond the prefix that aligns well against the path. For this reason, the path found is reconsidered to search its prefix that optimizes an alignment score. This alignment step finds the best extension sequence starting at the solid *k*-mer and obtaining the maximal alignment score. This extension problem is reminiscent of the best extension search for a local alignment in BLAST (which is solved with a drop-off score limit; Altschul *et al.*, 1990).

A note concerning an optimization of the inner region correction. When the path search between a source *k*-mer and all its targets has failed, it means that we cannot find a bridging path. Nevertheless, we can find the best extension on each side of the weak region and correct a prefix and a suffix of that region. For this, we use the same extension procedure as the head/tail correction, and adapt the graph path edge accordingly.

### 2.4 The graph path optimization

Finally, at the end of one complete pass of correction, all found inner paths have been recorded in the path graph. Here, an edge between two solid *k*-mers records the correction of the region dictated by the path found between those *k*-mers. Finally, after all inner solid *k*-mers have been considered, the correction of the inner region is optimized by finding a shortest path between the first and last solid *k*-mers of the read in the path graph using Dijkstra’s algorithm (Dijkstra, 1959).

### 2.5 Trimming and splitting corrected reads

In the end of the correction process, each base in a corrected read can be classified as solid if it belongs to at least one solid *k*-mer and weak otherwise. LoRDEC outputs the solid bases in upper case characters and weak bases in lower case. We provide two utilities for trimming and splitting the corrected reads. The first tool trims all weak bases from the beginning and the end of the reads but leaves intact regions of weak bases that are bordered by solid bases on both sides. Thus, one trimmed read is produced for each corrected read. The second tool both trims and splits the reads by extracting from the corrected reads all runs of solid bases as separate sequences.

## 3 RESULTS

### 3.1 Data and computing environment

We used three datasets of increasing size: one from *E.coli*, two eukaryotic ones from yeast and from the parrot. They include, respectively, 98 Mb, 1.5 and 6.8 Gb of PacBio reads, with 231, 451 Mb, and 35 Gb of Illumina reads. All details are given in Supplementary Table S1.

All experiments were run on servers with 16 cores operating at 2.53 GHz and 32 GB of memory. The runtimes were recorded with the Linux/Unix time command, and the memory and disk usage was recorded by polling the operating system periodically. Because all the correction tools support parallel execution on several cores, we report both total CPU time and elapsed (wall clock) time.

### 3.2 Evaluation approach

We used two approaches to evaluate the accuracy of correction. The first approach measures how well the reads align against the reference genome. The second approach compares the differences in the alignments of the original and corrected reads against the reference to evaluate the accuracy of correction.

For the *E.coli* and yeast datasets, we used BLASR (Chaisson and Tesler, 2012) to align the original and corrected reads to the genome and for the parrot dataset, we used BWA-MEM (Li, 2013). For the smaller datasets, BLASR was used because it tends to bridge long indels better and thus reports longer alignments. For the parrot dataset, we preferred BWA-MEM because it is faster. For each read, we kept its best alignment against the genome. We then counted the size of the aligned region of the reads, the size of the aligned regions in the genome and the number of identical positions in the alignments. The identity of the alignments was then calculated as the number of identical positions divided by the length of the aligned region in the genome. The reads corrected by an error correction program were then evaluated based on the size of the region that could be aligned against the genome, and the identity of the alignments.

The alignments of the original and corrected reads can be further analyzed to characterize the accuracy of correction. Consider a multiple alignment of the original read, the corrected read and the corresponding genomic region. Each position in this multiple alignment can be classified as true positive (TP), false positive (FP), true negative (TN) or false negative (FN). A position is TP if the original read has an error and it has been corrected by the error correction tool. Erroneous positions in the original read that have not been corrected are false negatives. In a FP position the error correction tool has made a correction, although there was no error in the original read, and finally, TN positions are correct in both original and corrected reads. The accuracy of correction can then be measured with several statistics:

- *Sensitivity* = TP/(TP + FN), how well does the tool recognize erroneous positions?
- *Gain* = (TP − FP)/(TP + FN), how well does the tool remove errors without introducing new ones?

Error Correction Toolkit (Yang *et al.*, 2013) is designed for comparing error correction results for second-generation sequencing data. As input, it requires the mapping of the original reads and of the corrected reads to the genome in SAM format. We used BLASR for the *E.coli* and yeast data and BWA-MEM for the parrot data to produce the alignments. For each pair of original and corrected read, the toolkit computes the set of differences with the reference genome, and it compares these two sets to determine TP, FP and FN positions with regard to correction. Whereas read mappers geared toward second-generation sequencing reads report full matches of the reads against the genome, BLASR and BWA-MEM report best local alignments of the reads against the genome. We modified the toolkit so that differences between original and corrected reads are counted only within the genomic region of the local alignment of the original read against the genome.

The comparison of the differences in alignments is not straightforward with large amounts of indels, as even the same differences can often produce different alignments with the same alignment score. Therefore, especially in partially corrected regions, more differences might be reported than is actually the case, and so this approach might report more FPs or FNs than are actually present in the datasets.

### 3.3 Effect of parameters on our approach

**Fig. 3.**
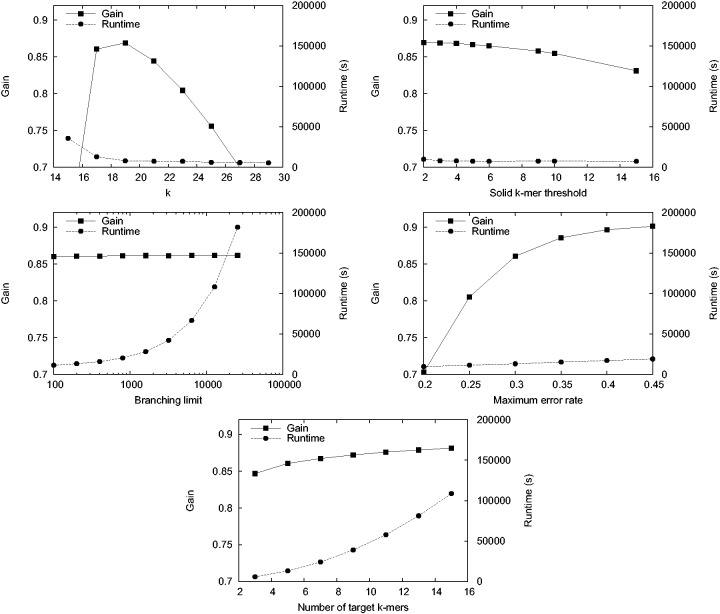
Effect of parameters on the runtime and gain of our method. We varied *k*, solid *k*-mer threshold, branching limit, maximum error rate and number of target *k*-mers one at a time, while other parameters were kept constant

We investigated the effect of parameters on our method on the *E.coli* dataset. We varied one parameter at a time and recorded the runtime and evaluated the accuracy of the method by computing gain. Figure 3 shows the results for this experiment when varying five parameters: the *k*, the threshold for a *k*-mer to be solid in the Illumina dataset, the maximum error rate of corrected regions, the branching limit and the number of target *k*-mers for path finding from a source *k*-mer. We see that *k* = 19 gives the best results for this dataset, and further experiments with the yeast data confirmed *k* = 19 to be optimal also for that dataset (data not shown). The solid *k*-mer threshold had only a modest effect on the accuracy of correction, a smaller threshold giving slightly better results. The accuracy of correction was improved by setting a higher maximum error rate of the corrected region with a slight increase in the runtime. Increasing the number of explored branches or the number of target *k*-mers had only a small effect on the gain, whereas runtime was increased considerably. Based on these observations, we ran our method on the *E.coli* and yeast data with the following parameters: *k*, 19; threshold for solid *k*-mers, 3; maximum error rate, 0.4; branching limit, 200; and number of target *k*-mers, 5. For the parrot data, we found *k* = 23 to give better results both in terms of runtime and accuracy. Supplementary Table S2 provides an explanation for each parameter and its default value.

### 3.4 Comparison against LSC and PacBioToCA

We compared LoRDEC against LSC (Au *et al.*, 2012) and PacBioToCA (Koren *et al.*, 2012). LSC was run with default parameters except that we set short read covered depth to the estimated coverage of the dataset, i.e. 50 for the *E.coli* dataset and 38 for the yeast dataset. PacBioToCA was run with default parameters except for tuning the parameters for parallelization to be suitable for our platform. The parameters for LoRDEC were set as explained above.

#### 3.4.1 Escherichia Coli

The runtime and memory and disk usage of the error correction tools are shown in Table 1 (top). LoRDEC is 17 times faster and requires 88% less memory and 95% less memory than LSC, which is the more resource efficient of the two previous tools on this dataset. The right side of Table 1 shows the correction statistics as reported by Error Correction Toolkit for LSC and LoRDEC, and we see that LoRDEC outperforms LSC.

**Table 1.** Runtime, memory, disk usage and accuracy statistics as reported by Error Correction Toolkit for the error correction tools on the *E.coli* (top), yeast (middle) and parrot (bottom) datasets

Data	Method	CPU time	Elapsed time	Memory	Disk	FP	TP	FN	Sensitivity	Gain
*E.coli*	PacBioToCA	45 h 18 min	3 h 12 min	9.91	13.59	NA	NA	NA	NA	NA
	LSC	39 h 48 min	2 h 56 min	8.21	8.51	695773	3149629	7845597	0.2865	0.2232
	LoRDEC	2 h 16 min	10 min	0.96	0.41	102427	9994561	1000665	0.9090	0.8997
Yeast	PacBioToCA^a^	792 h 41 min	21 h 57 min	13.88	214	NA	NA	NA	NA	NA
	LSC^b^	1200 h 46 min	130 h 16 min	24.04	517	7766700	38741658	80597251	0.3246	0.2596
	LoRDEC	56 h 8 min	3 h 37 min	0.97	1.63	2784685	100568850	18770059	0.8427	0.8194
Parrot	LoRDEC^b^	568 h 48 min	29 h 7 min	4.61	74.85	10591097	226996640	26296446	0.8962	0.8544

*Note*. Memory and disk usage are in gigabytes. The statistics could not be computed for reads corrected by PacBioToCA because PacBioToCA only reports trimmed and split reads. ^a^Run parallel on six servers. Memory usage is for one server. ^b^Run parallel on three servers. Memory usage is for one server.

The statistics of aligning the reads against the reference genome are shown in Table 2 (top). For LSC, we report the statistics both for the full corrected read set as reported by the tool and for the trimmed set. We note that LSC leaves out from the full read set any reads that it was not able to correct at all. Similarly, we report for LoRDEC statistics for full reads, reads trimmed at the ends and trimmed and split reads (see Section 2.5). LSC clearly performs worst of the three tools, whereas PacBioToCA and LoRDEC have similar statistics. Once corrected, trimmed and split by LoRDEC, the reads have slightly more bases, and a slightly smaller proportion of them align against the reference, but the identity of aligned regions is higher than for the reads corrected by PacBioToCA.

**Table 2.** Alignment statistics of the reads corrected by different tools on the *E.coli* (top), yeast (middle) and parrot (bottom) datasets

Data	Method	Size	Aligned	Identity	Genome coverage	
					Expected	Observed
*E.coli*	Original	1.0000	0.8800	0.9486	1.0000	0.9768
	PacBioToCA	0.7759	0.9965	0.9988	1.0000	0.9936
	LSC (full)	0.8946	0.9269	0.9579	1.0000	1.0000
	LSC (trim)	0.6824	0.9611	0.9725	1.0000	1.0000
	LoRDEC (full)	0.9318	0.8934	0.9952	1.0000	1.0000
	LoRDEC (trim)	0.8692	0.9419	0.9968	1.0000	1.0000
	LoRDEC (trim + split)	0.8184	0.9950	0.9997	1.0000	0.9979
Yeast	Original	1.0000	0.7900	0.9276	1.0000	0.9834
	PacBioToCA	0.7620	0.9887	0.9934	1.0000	0.9986
	LSC (full)	0.8760	0.8570	0.9420	1.0000	0.9988
	LSC (trim)	0.7020	0.9277	0.9544	1.0000	0.9992
	LoRDEC (full)	0.9771	0.8138	0.9741	1.0000	0.9995
	LoRDEC (trim)	0.9270	0.8492	0.9758	1.0000	0.9996
	LoRDEC (trim + split)	0.7412	0.9790	0.9928	1.0000	0.9984
	Original	1.0000	0.5060	0.9258	0.9235	0.8406
Parrot	LoRDEC (full)	0.9719	0.7633	0.9826	0.9769	0.9103
	LoRDEC (trim)	0.8423	0.8678	0.9838	0.9756	0.9085
	LoRDEC (trim + split)	0.7453	0.9782	0.9884	0.9773	0.9042

*Note*. The first column shows the ratio between the size of the read set and the original read set, the second column shows the ratio between the size of the aligned region of the reads and the size of the read set and the third column shows the alignment identity of the aligned regions. The last two columns give the expected and observed genome coverage by aligned reads, i.e. the proportion of the reference sequence covered by at least one read.

#### 3.4.2 Yeast

Both LSC and PacBioToCA failed to complete the correction of this dataset on a single server. We split the PacBio data and run LSC on three servers and PacBioToCA on six servers. Whereas PacBioToCA is designed to run distributed on several servers, LSC does not support distributed execution. Therefore, we chose to use as few servers as possible with LSC to minimize the effect of the distributed execution on the correction accuracy.

Table 1 (middle) shows the runtime, memory and disk usage statistics for the yeast dataset. Also for this dataset, LoRDEC uses at least one order of magnitude less time or memory and two orders less disk than PacBioToCA and LSC. For instance, LoRDEC is six times faster and uses 93% less memory and 99% less disk space than PacBioToCA. The gain and sensitivity of LSC remain <32%, while they stay >80% for LoRDEC. Table 2 (middle) compares the alignment statistics of the three tools: LSC (full or trim) aligns less bases with less identities than LoRDEC. PacBioToCA compared with LoRDEC (trim + split) yields slightly better alignments at a higher computational cost.

### 3.5 Experiments on the parrot data

We investigated the scalability of LoRDEC on a much larger eukaryotic dataset: the parrot data. As the parrot genome is a vertebrate, hence complex, genome, that is about one-third of the Human genome in length (Supplementary Table S1), it represents a real test for addressing both scalability and issues regarding the impact of genome organization. Given the running times of LSC and PacBioToCA on the smaller yeast data, these were not included in this experiment. The data contain three PacBio libraries, and we ran the correction of each on its own server. Table 1 (bottom) shows the runtime, memory and disk usage and statistics produced by Error Correction Toolkit. Based on these results, we can conclude that LoRDEC scales sufficiently to correct reads of large eukaryotic genomes on common computing hardware. The Error Correction Toolkit in Table 1 (bottom) and alignment statistics in Table 2 (bottom) show that the correction accuracy is comparable with the yeast dataset, although the reference is a draft genome containing more errors, and the alignment statistics also suffer from reads aligning to the end of scaffolds having only partial alignments.

### 3.6 Impact of the genome organization

**Fig. 4.**
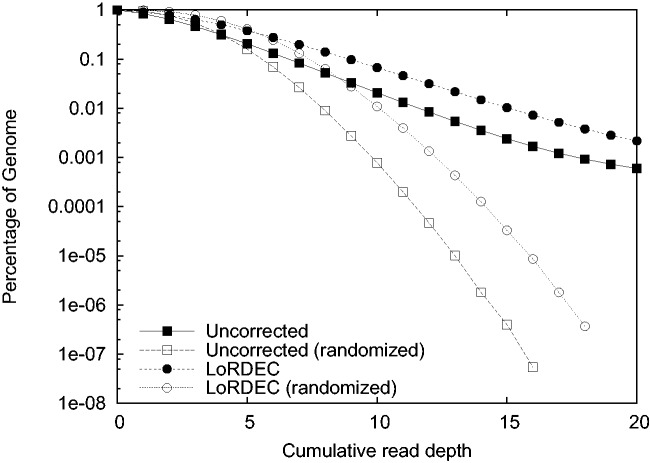
Percentage of the parrot genome covered by raw and corrected reads in function of read depth. The percentages (*y*-axis in log scale) are plotted for the true alignments (in black) and when considering the alignments are uniformly distributed over the genome (in white). Raw reads are represented by square and corrected reads by circles. The curves for corrected reads dominate that of raw reads, as correction increases the number of reads mapped. The black curves adopt similar shapes, suggesting that correction is not seriously impacted by repeats; their distances to the white curves suggest that a bias related to genomic location is already present in the raw reads

The evaluation of the correction delivered by LoRDEC indicates that it is accurate globally on all datasets. However, the genome organization, and especially the presence of repeats, could impact the quality of the correction. One could argue that in repeated regions, the solid k-mers and paths found in the DBG of SR may come from a different or from several copies of the repeat and mislead the correction process. In other words, the accuracy of the correction may vary along the genome. If this is the case, the distribution of reads with respect to the observed genome coverage should differ between raw and corrected reads. To assess this possibility, we computed the expected and observed genome coverages of the aligned raw and corrected reads (Last columns of Table 2). The coverage is computed as the number of genome positions covered by at least one alignment divided by the genome length. For the *E.coli* and Yeast case, the PacBio sequencing depth is in theory high enough to cover the whole genome (the expected coverage is one), and the effect of correction is to improve the observed coverage beyond 99%. Hence, no bias is visible in terms of coverage for these two cases. The case of the parrot data differs. First, the PacBio sequencing depth is only 5.5×, and thus about eight points separate the expected and observed coverages for both raw and corrected aligned reads (0.92 versus 0.84; 0.98 versus 0.90). To assess a possible bias, we plotted the percentage of the genome covered by aligned reads as a function of read depth for raw and corrected reads (black squares and circles in Fig. 4). We also plotted the same function but after randomizing the read positions, that is, as if the aligned reads where uniformly distributed over the whole genome (white squares and circles). First, both curves for real alignments depart from their randomized counterparts, showing that some bias exist in the genomic distribution of raw reads, but the same bias remains after correction. Various reasons may explain this bias including the low sequencing depth, locally wrong assembly or mapping bias. Second, the black curves have a similar shape, suggesting that the distribution in function of read depth is not affected by LoRDEC. Note that the curve of corrected reads remains above that of raw reads showing the improvement brought by LoRDEC at all read depths. Hence, even on a vertebrate genome, we conclude that LoRDEC can accurately correct PacBio reads with a small bias due to the genome organization.

## 4 CONCLUSION

Owing to their length, PacBio reads provide interesting information to connect other sequences, but this task is made considerably harder by their high error rate, which hinders alignment and similarity detection, both in terms of sensitivity and running time. As seen in our experiments, error correction with LoRDEC makes most of the sequence alignable with percentage of identity >97%. Previous correction programs achieve comparable accuracy, but with prohibitive computational resources. LoRDEC provides a significant improvement in this respect, to such a point that any genomics project can afford PacBio error correction, even with eukaryotic species. Moreover, hybrid error correction shall remain useful because it is powerful to combine distinct types of sequencing in a project.

Compared with other correction algorithms, LoRDEC offers a novel graph-based approach. Path searching in a DBG allows handling higher error rates. However, this search can fail if either no path or too many paths exist between the source and target *k*-mers. Some improvements seem reachable. When a path is missing, we plan to use the extension path search iteratively on each side of the inner region. A missing path may indicate a remaining adapter, and the local DBG structure could help identifying and removing it. In the case of too many paths, alternative values of *k* may help: a smaller *k* can introduce solid *k*-mers in the region and makes it shorter to solve. An algorithm to dynamically update the order (i.e. the parameter *k*) of the DBG would be useful in this respect (Cazaux *et al.*, 2014).

Additional experiments on PacBio RNA-seq reads show that LoRDEC could also improve the sequence of maize transcripts, which eased their alignment to a reference transcript database (see Supplementary data). LoRDEC is simple to use, scalable, can easily be incorporated in a pipeline and should adapt to other types of reads.

## Supplementary Material

Supplementary Data

## References

[btu538-B1] Altschul SF (1990). Basic local alignment search tool. J. Mol. Biol..

[btu538-B2] Au KF (2012). Improving PacBio long read accuracy by short read alignment. PLoS One.

[btu538-B3] Bashir A (2012). A hybrid approach for the automated finishing of bacterial genomes. Nat. Biotechnol..

[btu538-B4] Cazaux B (2014). From indexing data structures to de bruijn graphs. CPM, volume 8486 of LNCS.

[btu538-B5] Chaisson M (2004). Fragment assembly with short reads. Bioinformatics.

[btu538-B6] Chaisson MJ, Pevzner PA (2008). Short read fragment assembly of bacterial genomes. Genome Res..

[btu538-B7] Chaisson MJ, Tesler G (2012). Mapping single molecule sequencing reads using basic local alignment with successive refinement (BLASR): application and theory. BMC Bioinformatics.

[btu538-B8] Chikhi R, Rizk G (2012). Space-efficient and exact de bruijn graph representation based on a bloom filter. WABI, volume 7534 of LNCS.

[btu538-B9] Chin CS (2013). Nonhybrid, finished microbial genome assemblies from long-read smrt sequencing data. Nat. Methods.

[btu538-B10] Deshpande V (2013). Cerulean: a hybrid assembly using high throughput short and long reads. WABI, volume 8126 of LNCS.

[btu538-B11] Dijkstra EW (1959). A note on two problems in connexion with graphs. Numer. Math..

[btu538-B12] English AC (2012). Mind the gap: upgrading genomes with pacific biosciences rs long-read sequencing technology. PLoS One.

[btu538-B13] Gnerre S (2011). High-quality draft assemblies of mammalian genomes from massively parallel sequence data. Proc. Natl Acad. Sci. USA.

[btu538-B14] Koren S (2012). Hybrid error correction and de novo assembly of single-molecule sequencing reads. Nat. Biotechnol..

[btu538-B15] Li H (2013). Aligning sequence reads, clone sequences and assembly contigs with BWA-MEM.

[btu538-B16] Luo R (2012). SOAPdenovo2: an empirically improved memory-efficient short-read de novo assembler. Gigascience.

[btu538-B17] Navarro G, Mäkinen V (2007). Compressed full-text indexes. ACM Comput. Surv..

[btu538-B18] Pevzner PA (2001). An Eulerian path approach to DNA fragment assembly. Proc. Natl Acad. Sci. USA.

[btu538-B19] Philippe N (2013). CRAC: an integrated approach to the analysis of RNA-seq reads. Genome Biol..

[btu538-B20] Salikhov K (2013). Using cascading bloom filters to improve the memory usage for de brujin graphs. WABI, volume 8126 of LNCS.

[btu538-B21] Salmela L (2010). Correction of sequencing errors in a mixed set of reads. Bioinformatics.

[btu538-B22] Salmela L, Schröder J (2011). Correcting errors in short reads by multiple alignments. Bioinformatics.

[btu538-B23] Salzberg SL (2012). GAGE: a critical evaluation of genome assemblies and assembly algorithms. Genome Res..

[btu538-B24] Schröder J (2009). SHREC: a short-read error correction method. Bioinformatics.

[btu538-B25] Yang X (2013). A survey of error-correction methods for next-generation sequencing. Brief. Bioinform..

[btu538-B26] Zerbino DR, Birney E (2008). Velvet: algorithms for de novo short read assembly using de Bruijn graphs. Genome Res..

