# Supplementary material for: LoRDEC: accurate and efficient long read error correction
Source: Bioinformatics. 2014 Aug 26;30(24):3506–14. doi: 10.1093/bioinformatics/btu538 (PMC4253826; doi:10.1093/bioinformatics/btu538)
Supplement: Supplementary Data [file supp_30_24_3506__index.html]

LoRDEC: accurate and efficient long read error correction — LoRDEC: accurate and efficient long read error correction — LoRDEC: accurate and efficient long read error correction — Supplementary Data 

# LoRDEC: accurate and efficient long read error correction

## Supplementary Data

files

**Files in this Data Supplement:**

- Supplementary Data - pdf file
