# Supplementary material for: LoRDEC: accurate and efficient long read error correction
Source: Bioinformatics. 2014 Aug 26;30(24):3506–14. doi: 10.1093/bioinformatics/btu538 (PMC4253826; doi:10.1093/bioinformatics/btu538)
Supplement: Supplementary Data [file supp_btu538_acor-sm.pdf]

LoRDEC: Supplementary File: Data, Parameters, and Transcriptomes.

Leena Salmela and Eric Rivals

1 Data

	ECOLI	YEAST	PARROT
**Reference organism**			
Name	*Escherichia coli*	*Saccharomyces cerevisiae*	*Melopsittacus undulatus*
Strain	K-12 substr. MG1655	W303	NA
Reference sequence	NC_000913	CM001806-CM001823	BCM*
Genome size	4.6 Mbp	12 Mbp	1.23 Gbp
**PacBio Data**			
Accession number	DevNet[1]	DevNet[2]	ERR244164 ERR244165 ERR244166
Number of reads	33360	261964	4176242
Avg read length	2938	5891	1619
Max read length	14494	30164	16947
Number of bases	98 Mbp	1.5 Gbp	6.8 Gbp
Coverage	21x	129x	5.5x
**Illumina Data**			
Accession number	ERR022075[3]	SRR567755	ERR244156
Number of reads	2316613	4503422	345094538
Read length	100	100	101
Number of bases	231 Mbp	450 Mbp	35 Gbp
Coverage	50x	38x	28x

[1]https://github.com/PacificBiosciences/DevNet/wiki/E coli K12 MG1655 Hybrid Assembly
[2]https://github.com/PacificBiosciences/DevNet/wiki/Saccharomyces-cerevisiae-W303-Assembly-Contigs
[3]Only subset of data used

Table 1: Data sets used in the experiments evaluating the performance of error correction programs. The *E. coli* Illumina data set was truncated to 50x coverage to get shorter runtimes especially for LSC and PacBioToCA. For the yeast data Illumina reads were not available for the same sample as the PacBio reads so we chose a data set for the same yeast strain. For the parrot data set we used the highest ranking of the Assemblathon2 draft assemblies, BCM*, as the reference sequence [B+13].

2 Parameters impacting the correction process

Parameters & default		Explanation
-trials	5	Number of trials to find a path between the current k-mer and another solid k-mer downstream of a weak region. Increasing the number of trials also increases the number of found paths, which can only improve the correction, but also impacts the running time. A value between [5,9] yields good correction gain for a moderate impact on the running time.
-branch	200	This parameter limits the number of paths in the graph explored from each source k-mer. Our experiments show that good gain values in terms of correction are achieved for a parameter around a few hundreds, and that the gain remains stable. Increasing the branching limit by several orders of magnitude impacts strongly the running time, but does not yield large correction improvements, and is thus not recommended.
-errorrate	0.4	Estimation of the error rate in the PacBio reads. Surprisingly, the correction improves along with this estimate beyond most published estimations that lie [15-20]% error rates. Beyond the default value of 0.4 the correction stabilizes in our experiments.
-threads	1	A technical parameter controlling the maximum number of processes running at the same time for completing the correction. It allows to exploit parallel computation to improve the running time depending on the computer. It has no effect on the quality of the results.
<k-mer size>		The most crucial parameter. It depends on the PacBio sequencing error rate and on the target genome length. Its value should be within once and twice $\log_4(genome\_length)$. The lowest value in that range allowing many PacBio reads to have k-mers that also occur in short reads is appropriate. Those k-mers are likely solid and LoRDEC will attempt to find a correction path between those k-mers.
<abundance threshold>		If the number of occurrences of a k-mer is below this threshold in the short reads, the k-mer is said to be *weak*, otherwise it is *solid*. LoRDEC searches for a path either between two solid k-mers, or between a solid k-mer and one end of the long read. This filter resembles those performed during assembly for removing erroneous k-mers: so a threshold of 2 or 3 filters a majority of errors. The higher the threshold, the smaller the graph, the faster the correction. However, a too high threshold, say above 15% of the median coverage, will impact the sensitivity of the correction.

Table 2: Parameters of the correction program.

3 Correcting transcriptomic reads

We applied LoRDEC on the dataset of PacBio RNA-Seq reads from the B73 maize (Zea mays) transcriptome produced by Koren et al. [KSW+12] (Accession : SRX155708). To correct these long reads we used a HiSeq Illumina RNA-seq data from the same maize but from another library (Accession : SRR514100).

To assess the validity of the correction, we used BLAT [Ken02] with default parameters to align both the raw and the corrected reads to a reference database of cDNA sequences from maize. Before correction, only 110 Mbp of PacBio reads match the cDNA sequences, while this amounts to 232 Mbp for the corrected reads. Moreover, the alignments of corrected reads were split by BLAT into much fewer blocks (6 instead of 9 millions), reflecting the improved contiguity of their alignments. This experiment shows that LoRDEC can also correct RNA-seq long reads, thereby boosting their capacity to reliably match transcript sequences already present in reference databases.

3.1 Data for the maize

- cDNA reference data set version from April 26 2011, taken from PlantGDB (http://www.plantgdb.org/download/Download/xGDB/ZmGDB/ZMcdna.bz2)
- Illumina HiSeq data set: 97 million 151 nt long reads (http://sra.dnanexus.com/runs/SRR514100)
- PacBio read data set: 276715 reads (http://sra.dnanexus.com/experiments/SRX155708)

References

[B+13] K. R. Bradnam et al. Assemblathon 2: evaluating de novo methods of genome assembly in three vertebrate species. *GigaScience*, 2(1):1–31, 2013. 1

[Ken02] James W. Kent. BLAT—The BLAST-Like Alignment Tool. *Genome Res.*, 12(4):656–664, 2002. 3

[KSW+12] Sergey Koren, Michael C. Schatz, Brian P. Walenz, Jeffrey Martin, Jason T. Howard, Ganeshkumar Ganapathy, Zhong Wang, David A. Rasko, W. Richard McCombie, Erich D. Jarvis, and Adam M. Phillippy. Hybrid error correction and de novo assembly of single-molecule sequencing reads. *Nature Biotechnology*, 30(7):693–700, 2012. 3
